# AquaDesign: A tool to assist aquaculture production design based on abiotic requirements of animal species

**DOI:** 10.1371/journal.pone.0272508

**Published:** 2022-08-01

**Authors:** Grégoire Butruille, Marielle Thomas, Alain Pasquet, Nellya Amoussou, Lola Toomey, Axel Rosenstein, Sandrine Chauchard, Thomas Lecocq

**Affiliations:** 1 University of Lorraine, URAFPA, INRAE, Nancy, France; 2 LTSER France, Zone Atelier du Bassin de la Moselle, Vandœuvre-lès-Nancy, France; 3 University of Lorraine, AgroParisTech, INRAE, UMR Silva, Nancy, France; Swedish University of Agricultural Science, SWEDEN

## Abstract

Farming new species and promoting polyculture can enhance aquaculture sustainability. This implies to define the rearing conditions that meet the ecological requirements of a target species and/or to assess if different species can live in the same farming environment. However, there is a large number of rearing conditions and/or taxon combinations that can be considered. In order to minimise cumbersome and expensive empirical trials to explore all possibilities, we introduce a tool, AquaDesign. It is based on a R-script and package which help to determine farming conditions that are most likely suitable for species through *in silico* assessment. We estimate farming conditions potentially suitable for an aquatic organism by considering the species niche. We define the species n-dimensional niche hypervolume using a correlative approach in which the species niche is estimated by relating distribution data to environmental conditions. Required input datasets are mined from several public databases. The assistant tool allows users to highlight (i) abiotic conditions that are most likely suitable for species and (ii) combinations of species potentially able to live in the same abiotic environment. Moreover, it offers the possibility to assess if a particular set of abiotic conditions or a given farming location is potentially suitable for the monoculture or the polyculture of species of interest. Our tool provides useful pieces of information to develop freshwater aquacultures. Using the large amount of biogeographic and abiotic information available in public databases allows us to propose a pragmatic and operational tool even for species for which abiotic requirements are poorly or not available in literature such as currently non-produced species. Overall, we argue that the assistant tool can act as a stepping stone to promote new aquatic productions which are required to enhance aquaculture sustainability.

## Introduction

The world’s human population has increased dramatically since the 1950s, which has triggered a surge in the demand for food products [1]. This demand has been mainly fulfilled by upgrading already existing agricultural productions and developing new ones. Aquaculture is one of the agricultural sectors whose productions have skyrocketed these last decades [2]. The aquaculture contribution to total human-consumed aquatic products has increased from less than 5% in 1970 to more than 50% in 2018 [2]. Aquaculture is expected to take an even more prominent role in human food security and nutrition in the near future, especially as wild fisheries increasingly fail to meet the ever-growing demand for aquatic products [2]. Nevertheless, aquaculture is often criticised due to its negative environmental impacts and its potential unsustainability [3, 4]. Indeed, the development of aquaculture has triggered habitat destructions, biodiversity loss, biological invasions, and pathogen spill-overs, as well as water-quality degradation and eutrophication of wild aquatic ecosystems, notably due to a poorly efficient use of inputs and intrinsic resources of farmed systems [4]. Moreover, aquaculture production relies mainly on few species. For instance, only 27 species comprised over 90% of total finfish production in 2018 [2]. This low diversity of produced species jeopardises (i) human food security because the heavy dependence on few taxa puts at risk aquaculture production if, for instance, an epizootic outbreak happens in farmed species, and (ii) the future of productions since poorly diversified production limits the adaptive potential of aquaculture to face environment or consumer demand changes [5–7].

In order to overcome these issues, international organisations and scientists are strongly advocating for (i) production diversification in regard to species [7, 8] and (ii) application of agroecology concepts to minimise aquaculture environmental impacts, maximise the use of farming system resources, and promote the production of local species [4]. In this context, starting new domestication programs for currently unfarmed species and promoting development of polyculture (i.e. rearing/breeding two or more species in a particular production) have been pointed out as a way to design the tomorrow’s aquaculture [4, 8, 9].

Developing new species productions is challenging because it implies to use an appropriate farming environment that suits the species ecological requirements [9]. Identifying a suitable farming environment has often been based on trial-and-error approaches through empirical experimental attempts. However, such approaches are cumbersome and expensive. Therefore, they can be ineffective and inefficient since, for a particular species or species combination, there is a large number of farming environment set-ups that can be considered. Moreover, this can raise concerns about animal welfare (e.g. for fish) if unsuitable farming conditions are tested. This calls for the development of *in silico* approach that allows limiting the number of possibilities that should be assessed in subsequent experimental attempts.

The ecological niche is the match of a species to a specific set of environmental conditions [10]. The niche can be interpreted as a n-dimensional hypervolume in a space defined by several independent axes corresponding to species requirements (e.g. food size, temperature) [11]. The hypervolume boundaries indicate the conditions that permit the species growth and reproduction [11]. This interpretation has provided an operational concept to use species niches in applied sciences. Biological conservation is a stunning example of wide application of niche analyses to project consequences of habitat loss (e.g. [12]), climate change (e.g. [13]), or biological invasion (e.g. [14]), and to provide guidelines and recommendations to manage wild species and ecosystems (e.g. [15]). Similarly, ecological niche analyses can be regarded as a useful tool for development of new productions in aquaculture. Indeed, the ability of species to live in a particular farming environment and/or with other species could be evaluated by comparing the taxon niche with the farming environment and/or with niches of other species. Moreover, species niche analyses could provide useful pieces of information to design suitable farming environment from scratch. Nevertheless, such an approach has not been implemented in aquatic farming development. In order to move it to an operational approach for aquaculture development, an easy-to-use packaged workflow to estimate and compare species niches should be provided to aquatic farming stakeholders.

In this paper, we introduce an open source R-script and package [16] designed as an assistant tool, AquaDesign, to facilitate development of new animal species productions in monoculture and polyculture based on niche analyses [17, 18]. Our goal is to use the large amount of biogeographic and environmental information available in public databases to provide a pragmatic and operational way to highlight species or species combination that could be further considered in new aquaculture developments.

### Methodological and ecological assumptions to assess species niche

Several methods are available to define species niche, but most studies use a correlative approach where the species niche is estimated by relating distribution data to environmental conditions [19]. This approach is especially useful and pragmatic to estimate niches of large sets of species because it does not rely on time- and money-consuming experimental assessment in controlled conditions nor on detailed knowledge about ecological requirements of a particular species. Moreover, it relies on dataset mined from increasingly available ecological databases.

Using species niche for aquaculture purpose implies first to determine which environmental variables must be considered as relevant for aquatic species. These relevant variables can be different between marine and freshwater species. Since current aquaculture is dominated by inland productions [2], we designed our assistant tool for freshwater production development and focused on variables relevant for this specific production. These latter encompass biotic and abiotic variables. Although biotic variables are important shaping factors of species niche, the primary condition for a taxon to be reared in a particular farming environment is its ability to withstand the abiotic conditions. Regardless of biotic components, any aquaculture environment cannot be regarded as potentially suitable for a target species if it does not match with the taxon abiotic requirements. Therefore, we focused on abiotic variables to develop our assistant tool.

In order to select abiotic variables for our assistant tool, we first established an initial list of relevant variables to be considered for various freshwater aquaculture systems (i.e. pond, raceway, recirculating aquaculture system, pen, and cage) according to scientific literature about ecological and physiological requirements of aquatic animal species (e.g. [20–25]) (S1 Table). For all of these variables, a temporal variation occurs in the wild (i.e. seasonality). Therefore, we selected variables reflecting these variabilities (e.g. maximum temperature of the warmest month and minimum temperature of the coldest month). Then, we restricted the initial list according to the data availability of (i) each abiotic variable or (ii) proxy allowing inference of an abiotic variable (Table 1). Since correlative approach implies overlaying occurrence records with locally observed environmental variables, we chose to use only variables for which datasets can be mined from ecological databases with a worldwide coverage (Table 1).

**Table 1:** Abiotic variables considered in the assistant tool.

Variable	Unit	Variable description and/or justification	Source
Annual upstream air temperature	°C*10	Proxy for water temperature [26].	Earthenv (WorldClim)
Maximum air temperature of the warmest month	°C*10	Maximum temperature observed in the species’ distribution area.	Earthenv (WorldClim)
Minimum air temperature of the coldest month	°C*10	Minimum temperature observed in the species’ distribution area.	Earthenv (WorldClim)
Upstream air temperature annual range	°C*10	Temperature long-term variability in the species’ distribution area.	Earthenv (WorldClim)
Mean air temperature of the driest quarter	°C*10	Temperature when the thermal inertia is supposed to be minimal.	Earthenv (WorldClim)
Maximum pH of the soil	pH*10	Proxy for the pH of water.	Earthenv (ISRIC)
Annual upstream precipitation	mm	Proxy for parameters with varying concentrations (Ammonia, nitrate…).	WorldClim
		Can affect water pH due to runoff or acid rainfalls. Controls water availability in ponds (floods and droughts) and water conductivity.	
		Effects on turbidity [25].	
Upstream precipitation of wettest month	mm	Maximum precipitation observed in the species’ distribution area during the wettest month.	WorldClim
Upstream precipitation of driest month	mm	Maximum precipitation observed in the species’ distribution area during the driest month.	WorldClim
Slope average	[°] *100	Proxy for flow, water current, and dissolved oxygen concentration.	Earthenv (HydroSHEDS)
Annual average solar radiation	kJ.m^-2^.day^-1^		WorldClim
Annual average water vapor pressure	kPa		WorldClim
Minimum daylength	Hours	Minimum sunshine duration observed in the species’ distribution area.	geosphere
Maximum daylength	Hours	Maximum sunshine duration observed in the species’ distribution area.	geosphere
Daylength range	Hours	Photoperiod range in the species’ distribution area.	geosphere
Average elevation	m	Proxy for temperature, pressure (and dissolved oxygen concentration), streamflow…	Earthenv (HydroSHEDS)
Average annual flow	m^3^.s^-1^	Proxy for water renewal.	FLO1K
Maximum annual flow	m^3^.s^-1^	Maximum water flow observed in the species’ distribution area.	FLO1K
Minimum annual flow	m^3^.s^-1^	Minimum water flow observed in the species’ distribution area.	FLO1K

### Baseline datasets and data preparation

Abiotic datasets are obtained from three databases, EarthEnv [26], FLO1K [27], and WorldClim [28], as well as from one R-package, *geosphere* [29] (Table 1). Occurrence records are mined from the online Global Biodiversity Information Facility (GBIF, http://www.gbif.org) using the R-package *rgbif* [30]. The assistant tool allows users to select which species is/are downloaded by indicating species taxonomic names and returns a proposed correction in case of misspelling based on the GBIF taxonomical baseline. Occurrence data quality issues are minimised [31, 32] by removing potential spatial errors in GBIF using the R-package *CoordinateCleaner* [33]. It eliminates suspicious data (e.g. isolated points, mismatch between coordinates and country code, see discarding criteria in [33]). Since our tool is designed for freshwater aquaculture, points located in the sea are removed.

Characterising niche by correlative approach requires that all occurrence and abiotic datasets share a same coordinate system (WGS84), spatial extent, and geographic scale. Therefore, our R-script limits spatial extent to 56°S to 60°N and 145°W to 180°E, which is the current coverage of EarthEnv. Then, it aggregates datasets at the same resolution grid by (i) recording only species absence/presence per grid cell to account for differences in local sampling effort and to obtain reliable absence data [34] and (ii) merging abiotic dataset by calculating the mean of each abiotic variable within each grid cell, except for daylength values for which the value at the cell centre is attributed to the whole cell. The user can choose to rescale the data either at 10 or 30 arcminutes (~18 or ~55 km at the equator). It is worth noting that the highest resolution might be more precise, but also increases the risk of generating false absence data, which could bias assistant tool results [35]. We recommend using 10 arcminutes only for species with large occurrence point datasets.

## Data processing according to user’s objectives

### Assessing potential suitability of an available farming environment

Assessing whether a particular farming environment is potentially suitable for a species of interest can be done by checking that abiotic conditions of this environment are included in the species hypervolume. The assistant tool allows performing this assessment for aquaculture facilities relying on local abiotic conditions (i.e. outdoor aquaculture) through three steps. First, species niche hypervolume is computed using the support vector machine method, which is appropriate when dimensionality is high as in our abiotic datasets (R-package *hypervolume*, [36]). Nevertheless, the method accuracy can be negatively impacted if the dimension number exceeds the log of occurrence sum [36]. Therefore, the assistant tool reduces the dimensionality of the initial dataset while minimising collinearity between variables through a principal component analysis (PCA) in which only dimensions with an eigenvalue superior to 1 are retained (i.e. Guttman-Kaiser criterion). Nevertheless, despite this dimensionality reduction, if the dimension number still exceeds the log of occurrence sum, the assistant tool returns a warning to users. During the PCA processing, abiotic variables are standardised. Second, the abiotic conditions corresponding to a given farming location are obtained by extracting the abiotic conditions occurring at the farming location (point or area) with the same procedure as the one to match species occurrences with local abiotic variable values. Alternatively, the user can directly provide values of abiotic variables, a useful option if farming conditions are regulated by farmers and modified from those occurring locally (e.g. use of thermal water from power plant for farming). Third, an inclusion test checks whether the projection of the given farming location/system in the n-dimensional space is included in the species niche hypervolume (R-package *hypervolume*, [36]).

### Assisting definition of new farming environment from scratch

When designing a new farming environment for a species from scratch is aimed, knowing abiotic conditions occurring across the distribution range of this species can facilitate the design process. However, a species can undergo quite different abiotic conditions across its distribution range, especially for widespread taxa. This can shape geographic differentiation in abiotic requirements between conspecific populations due to local adaptations (e.g. [37]). This raises the question of which abiotic conditions should be applied for production of a target species. The choice of these conditions will depend on strategy used to create the initial organism stock that will be used in production. Two alternative paradigms are commonly used to create this stock [38, 39]: (i) using only one population or (ii) mixing several geographically distant populations. For the former, the assistant tool can provide the abiotic conditions occurring at the population location. For the latter, we consider that the most commonly observed values of each abiotic variable across the species range are those which are the most likely suitable conditions for most of the populations that will be mixed. The assistant tool highlights these commonly observed conditions by displaying density diagrams for each variable. These diagrams are obtained using the geom_density function from the R-package *ggplot2* [40].

### Assessing polyculture potential feasibility

The ability of several species to live in the same abiotic environment can be assessed through the intersection between species niche hypervolumes: species with non-null intersection can be potentially co-farmed. Nevertheless, larger intersect means that species can be co-farmed in a larger set of abiotic conditions. This makes their polyculture (i) feasible in a larger set of farming conditions and (ii) still possible in the event of abiotic variations in the farming environments (e.g. extreme climatic events in ponds). Therefore, the assistant tool allows (i) computing the intersection hypervolume between species and (ii) ranking all tested combinations according to the extent of the intersection hypervolume. Since the R-package *hypervolume* [36] did not allow comparing more than two hypervolumes at once, we designed the function hypervolume_set_n_intersection to compare n species. The assistant tool also provides the possibility for users (i) to show the overlap of density diagrams of all species of a particular combination to highlight common suitable abiotic parameters or (ii) to assess that a particular farming facility/location is included in the intersection hypervolume according to procedures detailed previously.

### Test cases

We assessed if the assistant tool achieves its intended purpose through *in silico* evaluation of already known instances of polyculture.

Firstly, we selected as test cases three scientific references in which authors studied polyculture in an outdoor aquaculture production (i.e. ponds and cages) at a particular location. Test case 1: a pond system with a polyculture of common carp (*Cyprinus carpio*), roach (*Rutilus rutilus*), and tench (*Tinca tinca*) located at 48.1203°N, 1.7925°W in France [41]. Test case 2: ponds with a polyculture of catla (*Catla catla*), common carp (*C*. *carpio*), grass carp (*Ctenopharyngodon idella*), Java barb (*Barbonymus gonionotus*), mrigal (*Cirrhinus mrigala*), rohu (*Labeo rohita*), and silver carp (*Hypophthalmichthys molitrix*) in an area located at 23.7000⁰N– 23.9833⁰N, 88.9167⁰E– 89.0667⁰E in Bangladesh [42]. Test case 3: earthen ponds with polyculture of African sharptooth catfish (*Clarias gariepinus*) and Nile tilapia *(Oreochromis niloticus*) in an area located at 1.1667⁰S– 1.6000⁰S, 34.1333⁰E– 35.0167⁰E in Tanzania. Each test case is hereafter analysed independently. To our knowledge, farming locations of the test cases have not been recorded as species occurrences in GBIF. Therefore, we considered these polyculture farming locations as an effective external dataset to validate the relevance of the assistant tool.

Secondly, we assessed using the assistant tool if species of each test case can be potentially co-farmed. This assessment is based on intersections between the niche hypervolumes of all species from the same test case. We obtained occurrence records from GBIF for each species and abiotic datasets from EarthEnv, FLO1K, WorldClim, and the R-package geosphere (databases accessed on January 22, 2022). Potential occurrence data quality issues were fixed with the R-package CoordinateCleaner. For each species, the number of occurrences were (before/after fixing data quality issues): *B*. *gonionotus* = 508/69; *C*. *catla* = 23/0; *C*. *mrigala* = 426/41; *C*. *gariepinus* = 4,461/4,140; *C*. *idella* = 7,459/7,133; *C*. *carpio* = 179,600/170,826; *H*. *molitrix* = 5,230/5,179; *L*. *rohita* = 640/56; *O*. *niloticus* = 5,188/4,721; *R*. *rutilus* = 706,001/536,114; *T*. *tinca* = 145,701/133,242. We thus excluded *C*. *catla* from the test case 2. Then, we aggregated datasets at the 30 arcminutes resolution. We computed species niche hypervolume after reducing with a PCA and keeping only dimensions with an eigenvalue superior to 1. Since three species (*B*. *gonionotus*, *C*. *mrigala*, and *L*. *rohita*) from the test case 2 had still a dimension number exceeding the log of occurrence sum, we excluded them from the assessment. Then, in each test case, we computed the intersection between niche hypervolumes of all species. For all test cases, the intersection between all species niche hypervolumes was not null, meaning that polyculture of analysed species is feasible according to our approach.

**Fig 1:**
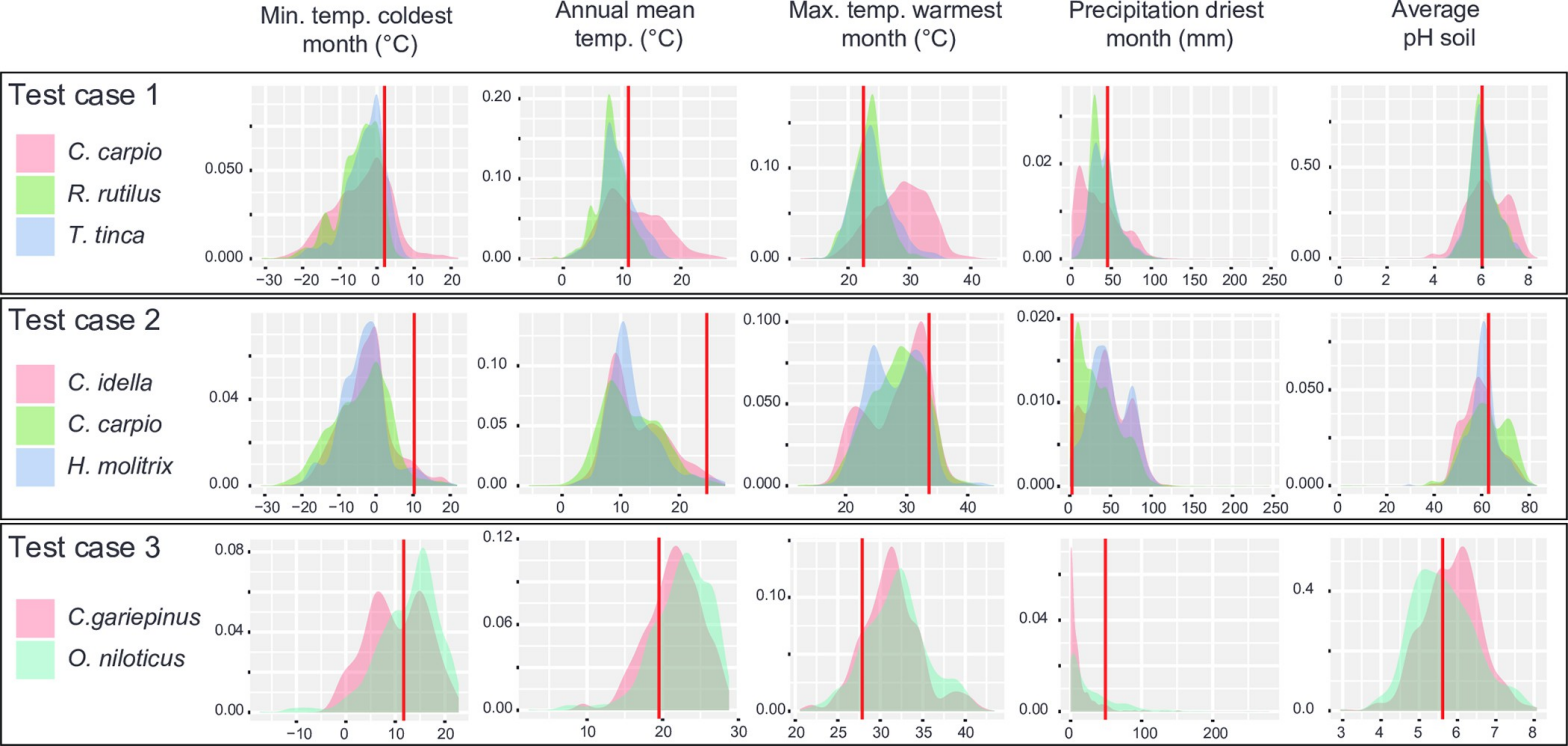
Density plots showing the distribution of a set of abiotic parameters based on occurrences used to determine species niche hypervolumes for the three test cases. Abiotic parameters are in columns: Min. temp. coldest month = Minimal temperature of the coldest month; Annual mean temp. = Annual mean temperature; Max. temp. warmest month = Maximal temperature of the warmest month; Precipitation driest month = Precipitation of the driest month; Average pH soil = Average pH observed in the soil. Other abiotic parameters were considered by the assistant tool (see Table 1), but only a subset of abiotic parameters is shown in the figure. Test cases are in lines. For each test case, a colour chart identifies density diagrams of each analysed species. The analysed species are *Clarias gariepinus*, *Ctenopharyngodon idella*, *Cyprinus carpio*, *Hypophthalmichthys molitrix*, *Oreochromis niloticus*, *Rutilus rutilus*, and *Tinca tinca*. The red lines correspond to position of the farming site of each test case in abiotic density plots.

Thirdly, we assessed using the assistant tool if the different polycultures can be performed at locations of each test case. We obtained the abiotic conditions at location of each farming place based on dataset mined from EarthEnv, FLO1K, WorldClim, and the R-package geosphere (databases accessed on January 22, 2022). Since we chose a 30 arcminutes resolution, the used coordinates of farming location were: test case 1 = 48.25°N; -1.75°E; test case 2 = 23.75°N; 88.75°E; test case 3 = -1.25°N; 34.75°E. Then, we performed inclusion tests to assess if each given farming location is included in the intersection hypervolume between all analysed species of each test case. For each test case, farming location was included in the intersection hypervolume. This means that already known instances of polyculture are considered as feasible at their specific location according to the assistant tool. Finally, we displayed the density diagrams of all analysed species for key abiotic parameters along with the position of the farming site in these diagrams for each analysed species of each test case (Fig 1).

### Limits and prospects

Overall, three main limitations of our assistant tool should be stated.

Firstly, although GBIF hosts occurrence datasets for several tens of thousands of fish species (*sensu* all taxa included in Myxini [hagfishes], Cephalaspidomorphi [lampreys], Holocephali [chimaeras], Elasmobranchii [sharks and rays], Sarcopterygii [lobe-finned fishes], and Actinopterygii [ray-finned fishes]), the quantity of information is uneven across species (see test cases). Special attention should be paid to quantity, distribution, and density of occurrences available for each species prior to using the assistant tool. Indeed, low occurrence dataset quality can result in biased assessment for the three here above described method applications. Nevertheless, assuming that the assistant tool will be mainly used for species of interest, which have thus been the focus of an abundant research, quantitative and qualitative datasets is likely to be available for users.

Secondly, using correlative approach for niche analyses allows estimating realised niche (i.e. all the environmental conditions where the species currently lives) rather than fundamental niche (i.e. the full range of environmental conditions in which a species is able to live, without any other limiting factors that could constrain the species distribution). Therefore, we cannot rule out that assistant tool results would be too restrictive due to an underestimation of species fundamental niche. However, determining fundamental niche is still hard-to-achieve without detailed knowledge on species (e.g. [43]). We argue thus that the realised niche remains the most operational approach to propose a pragmatic tool for aquaculture development.

Finally, we designed our assistant tool according to worldwide biogeographic and abiotic databases currently available. It implied to (i) discard potentially key abiotic variables (S1 Table) due to their poor availability at large geographic scale or to (ii) use proxies which are only partially correlated to key variables. This can potentially decrease the relevance of our approach. However, we speculate that future development of databases will make available more abiotic variables which will be integrated in our approach and will reinforce the relevance of assessment performed by our assistant tool.

The current version of our assistant tool mainly allows assessing or designing farming environments relying on local abiotic conditions for freshwater productions. However, it can be applied to facilitate the development of indoor highly human-controlled systems (e.g. indoor recirculated aquaculture systems) or marine species productions provided that the list of abiotic variables is adjusted to the farming system specificities (e.g. altitude is not a relevant proxy for marine production development) and to the taxon group. Beside aquaculture purpose, our assistant tool could be applied for further purposes such as *ex-situ* conservations of aquatic species in order to design hosting facilities for endangered taxa.

### Availability

The current stable version of the package requires R 4.1.1 and can be downloaded from github (https://github.com/GregoireButruille/AquaDesign). The R-script is available in figshare [17]. A tutorial of the assistant tool is available in figshare [18].

## Supporting information

S1 Table Abiotic component selected as relevant considering environmental requirements and physiology of aquatic organisms. (XLSX)
